# Supplementary material for: Distinct subpopulations of ventral pallidal cholinergic projection neurons encode valence of olfactory stimuli
Source: bioRxiv. 2023 Oct 10:2023.10.06.561261. Preprint. [Version 1] doi: 10.1101/2023.10.06.561261 (PMC10659428; doi:10.1101/2023.10.06.561261)

bioRxiv preprint doi: https://doi.org/10.1101/2023.10.06.561261; this version posted October 10, 2023. The copyright holder for this preprint (which was not certified by peer review) is the author/funder. This article is a US Government work. It is not subject to copyright under 17 USC 105 and is also made available for use under a CC0 license.

Supplementary Figure Legends

Figure S1 (supplementary to Fig 1): Odor induced approach and avoidance behaviors are not due to odor induced changes in locomotor activity. Neither the appetitive odor (APP) nor the aversive odor (AV) significantly altered **(A)** total distance traveled or **(B)** velocity during the odor preference test vs. a null odor stimulus (N, saline diluent).

Figure S2 (supplementary to Fig 2): Workflow of the analysis pipeline for the detection of activated VP cholinergic neurons.

A. Immunohistochemistry for Substance-P was conducted in a Chat-tau-eGFP mouse to assist in delineating VP borders.

B. Following the odor preference test, tissue was collected and processed for (IHC) for ChAT (to mark cholinergic neurons) and cFos (to label activated neurons). A whole slice image was acquired using the Olympus VS200 slide scanner (20x objective, minimal exposure times for 405/488/594/647 nm channels). Files were converted to Imaris files and uploaded to Imaris software for quantification.

C. The whole slice image is cropped and masked to contain only signal within the VP.

D. A signal-based intensity and diameter threshold is set using the spots function in Imaris to detect cFos signal. This threshold is then used to create a new masked channel with only the cFos signal (Left: raw cFos signal, Middle: threshold for cFos signal, Right: masked cFos signal).

E. The surfaces function in Imaris was used to set a signal-based intensity for ChAT detection. This threshold is then used to create a new masked channel containing only ChAT signal (Left: raw ChAT signal, Middle: threshold for ChAT signal, Right: masked ChAT signal)

F. Imaris is used to detect the colocalized pixels of the two masked channels.

Figure S3 (supplementary to Fig 4): Chemogenetic inhibition of VP cholinergic neurons induce changes in odor preference but not changes in locomotor activity.

To assess the effects of the inhibition of VP cholinergic neurons on approach and/or avoidance behaviors, Chat-Cre mice were injected with AAV.hSyn.DIO-hM4Di and AAV.Syn.eGFP (hM4Di experimental group) or AAV.Syn.eGFP only (Sham) in the VP. Following recovery from surgery, all mice were injected IP with 0.1 mg/kg clozapine 15-minutes prior to an odor preference test in a Y-Maze. In the odor preference test, mice were allowed access to two arms of the Y-Maze (appetitive (APP) odor vs. saline **(A & B)** or aversive (AV) odor vs. saline **(C & D)**). Time spent in each arm, as well as locomotor activity (distance traveled and velocity) were assessed.

Top = DIO-hM4Di APP odor preference test. There is no difference between mice in the sham group and mice that express DIO-hM4Din **(A)** distance traveled or **(B)** velocity during the APP odor preference test.

Bottom = DIO-hM4Di AV odor preference test. There is no difference between mice in the sham group and mice that express DIO-hM4Di in **(A)** distance traveled or **(B)** velocity during the AV odor preference test.

bioRxiv preprint doi: https://doi.org/10.1101/2023.10.06.561261; this version posted October 10, 2023. The copyright holder for this preprint (which was not certified by peer review) is the author/funder. This article is a US Government work. It is not subject to copyright under 17 USC 105 and is also made available for use under a CC0 license.

<u>Figure S4 (supplementary to Fig 5 & 6):</u> The order of odor presentation does not affect the total number of VP cholinergic neurons activated.

Odor exposure (either appetitive odor (APP) or aversive odor (AV)) significantly increases the number of activated VP cholinergic neurons compared to a null odor stimulus (N, saline diluent). Regardless of order of odor presentation (i.e., (**A**) Day 1 vs. (**B**) Day 2) and method in which activated VP cholinergic neurons are assayed (i.e., (**A**) ADCD vs. (**B**) ChAT and cFos IHC), both odors (APP or AV) significantly increase the number of activated VP Cholinergic neurons vs. a null odor stimulus. * $p < 0.05$.

<u>Figure S5 (supplementary to Fig 5 & 6):</u> Experiments using the robust activity marker (RAM) confirm ADCD and cFos labeling experiments.

A. A distinct activity-dependent viral vector was used to verify results from ADCD and cFos labeling experiments. The robust activity marker (RAM) viral vector utilizes a synthetic activity-dependent promoter and a Tet-Off system to label activated neurons. RAM was injected in the VP of wild-type C57/BL6J mice. The RAM construct was used in conjunction with ChAT and cFos IHC to label activated VP cholinergic neurons in two distinct contexts. The behavioral paradigm used was identical to the protocol used for ADCD and cFos labeling experiments. Representative images from RAM experiments showing RAM positive (activated neurons on Day 1), cFos positive (activated cells on Day 2), ChAT (cholinergic marker), and the colocalization of RAM + ChAT and cFos + ChAT.

B. & C. Confirming results from the ADCD and cFos labeling experiments (in Fig 6), mice exposed to a different odor on Day 2 exhibited no overlap between ChAT+/RAM+ and ChAT+/cFos+ neurons.

<u>Figure S6 (supplementary to Fig 5 & 6):</u> ADCD and RAM are comparable in labeling activated VP cholinergic neurons.

For ADCD experiments, Chat-Cre x Fos-tTA/GFP mice were injected with ADCD in the VP. For Test 1, ADCD was used to assess the number of activated VP cholinergic neurons. On Test 2, the colocalization of ChAT and cFos-GFP (examined using IHC) was used to assess the number of activated cholinergic neurons (see Fig 5 and Fig 6 for details). For RAM experiments, WT mice were injected with RAM in the VP. For Test 1, the number of RAM+ neurons co-labeled with ChAT IHC was used to assess the number of activated VP cholinergic neurons. For Test 2, the colocalization of ChAT and cFos (examined using IHC) was used to assess the number of activated VP cholinergic neurons (see Fig S5 for details). Regardless of order of odor presentation (Left = appetitive odor (APP), Right = aversive odor (AV)), ADCD and RAM labeled similar number of activated VP cholinergic neurons.

<u>Figure S7 (supplementary to Fig 7):</u> ADCD-hM4di induced changes in odor preference are not due to changes in locomotor activity.

To assess the effects of the inhibition of previously activated VP cholinergic neurons on approach and/or avoidance behaviors, Chat-Cre x Fos-tTA/GFP mice were injected with ADCD and AAV.Syn.eGFP (experimental group), or AAV.Syn.eGFP only (Sham) in the VP (see Fig 10 legend and methods for details). Following recovery from surgery, mice were habituated in the Y-Maze (2 x 10 min) and taken off a DOX diet. Approximately 24-hours later, mice were exposed to an odor (either appetitive (APP) or aversive (AV)) in one arm of the Y-Maze.

bioRxiv preprint doi: https://doi.org/10.1101/2023.10.06.561261; this version posted October 10, 2023. The copyright holder for this preprint (which was not certified by peer review) is the author/funder. This article is a US Government work. It is not subject to copyright under 17 USC 105 and is also made available for use under a CC0 license.

Following odor exposure, mice were placed on a DOX diet to prevent further expression of ADCD. Approximately 24-hours later, all mice were injected IP with 0.1 mg/kg clozapine 15-minutes prior to an odor preference test in a Y-Maze. In the odor preference test, mice were allowed access to two arms of the Y-Maze (previously exposed odor, either APP or AV, vs. saline). Time spent in each arm, as well as locomotor activity (distance traveled and velocity) were assessed.

Top = ADCD-hM4Di appetitive (APP) odor preference test. There is no difference between mice in the sham group and mice that express ADCD-hM4Di in **(A)** distance traveled or **(B)** velocity during the APP odor preference test.

Bottom = ADCD-hM4Di aversive (AV) odor preference test. There is no difference between mice in the sham group and mice that express ADCD-hM4Di in **(C)** distance traveled or **(D)** velocity during the AV odor preference test.

<u>Figure S8 (supplementary to Fig 9):</u> Comparison of the electrophysiological properties of appetitive (APP) vs. AV (AV) VP cholinergic neurons demonstrates that they are largely similar to one another and to the overall population of VP cholinergic neurons (see Fig 8 for differences).

The majority of both passive and active membrane properties are the same whether the recordings are from APP or AV activated VP cholinergic neurons. Similarities between the two are observed in **(A)** input resistance, **(B)** tau, **(C)** threshold, **(D)** rheobase, **(E)** action potential amplitude, **(F)** action potential width, **(G)** action potential upstroke, **(H)** actional potential downstroke, **(I)** sag potential, **(J)** afterhyperpolarization latency, **(K)** afterhyperpolarization width, **(L)** coefficient variation**,** **(M)** frequency-current slope, **(N)** adaptation index and **(O)** max firing.

Both APP and AV activated VP cholinergic neurons significantly differ from ALL VP cholinergic neurons in **(F)** action potential width and **(I)** sag potential.

APP odor activated VP cholinergic neurons significantly differ from ALL VP cholinergic neurons in **(A)** input resistance, **(D)** rheobase, **(M)** frequency-current slope, **(N)** adaptation index and **(O)** max firing.

AV odor activated VP cholinergic neurons significantly differ from ALL VP cholinergic neurons in **(J)** afterhyperpolarization latency.

<u>Figure S9 (supplementary to Figure 10):</u> Additional morphological properties between appetitive odor activated (APP) and aversive odor activated (AV) VP cholinergic neurons.

A. Assay of the number of 1° , 2° , and 3° dendrites revealed statistically significant differences between APP vs. AV VP cholinergic neurons for only 2nd order dendrites. There was no significant difference between groups in the number of primary or tertiary dendrites. The number of secondary dendrites was significantly higher in appetitive odor (APP) vs. aversive odor (AV) activated VP cholinergic neurons (* $p < 0.05$).

Despite differences in proximal complexity (see Fig 9), there was no significant differences between groups in **(B)** branching angle, **(C)** mean dendrite length, **(D)** number of dendrite branch points or **(E)** number of dendrite terminal points.

bioRxiv preprint doi: https://doi.org/10.1101/2023.10.06.561261; this version posted October 10, 2023. The copyright holder for this preprint (which was not certified by peer review) is the author/funder. This article is a US Government work. It is not subject to copyright under 17 USC 105 and is also made available for use under a CC0 license.

Supplementary Figures

bioRxiv preprint doi: https://doi.org/10.1101/2023.10.06.561261; this version posted October 10, 2023. The copyright holder for this preprint (which was not certified by peer review) is the author/funder. This article is a US Government work. It is not subject to copyright under 17 USC 105 and is also made available for use under a CC0 license.

Fig S1

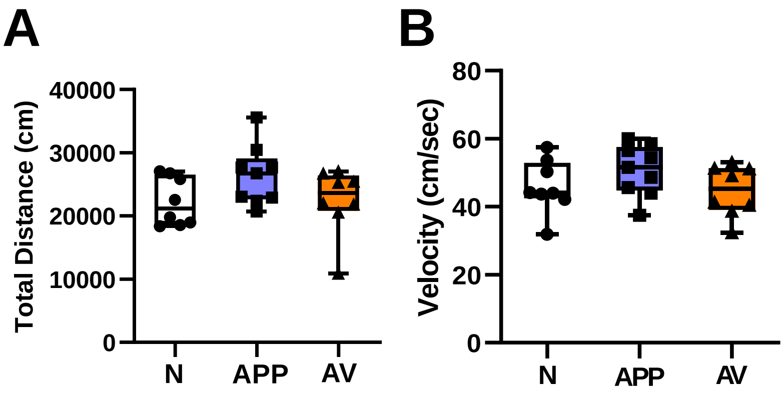

bioRxiv preprint doi: https://doi.org/10.1101/2023.10.06.561261; this version posted October 10, 2023. The copyright holder for this preprint (which was not certified by peer review) is the author/funder. This article is a US Government work. It is not subject to copyright under 17 USC 105 and is also made available for use under a CC0 license.

Fig S2

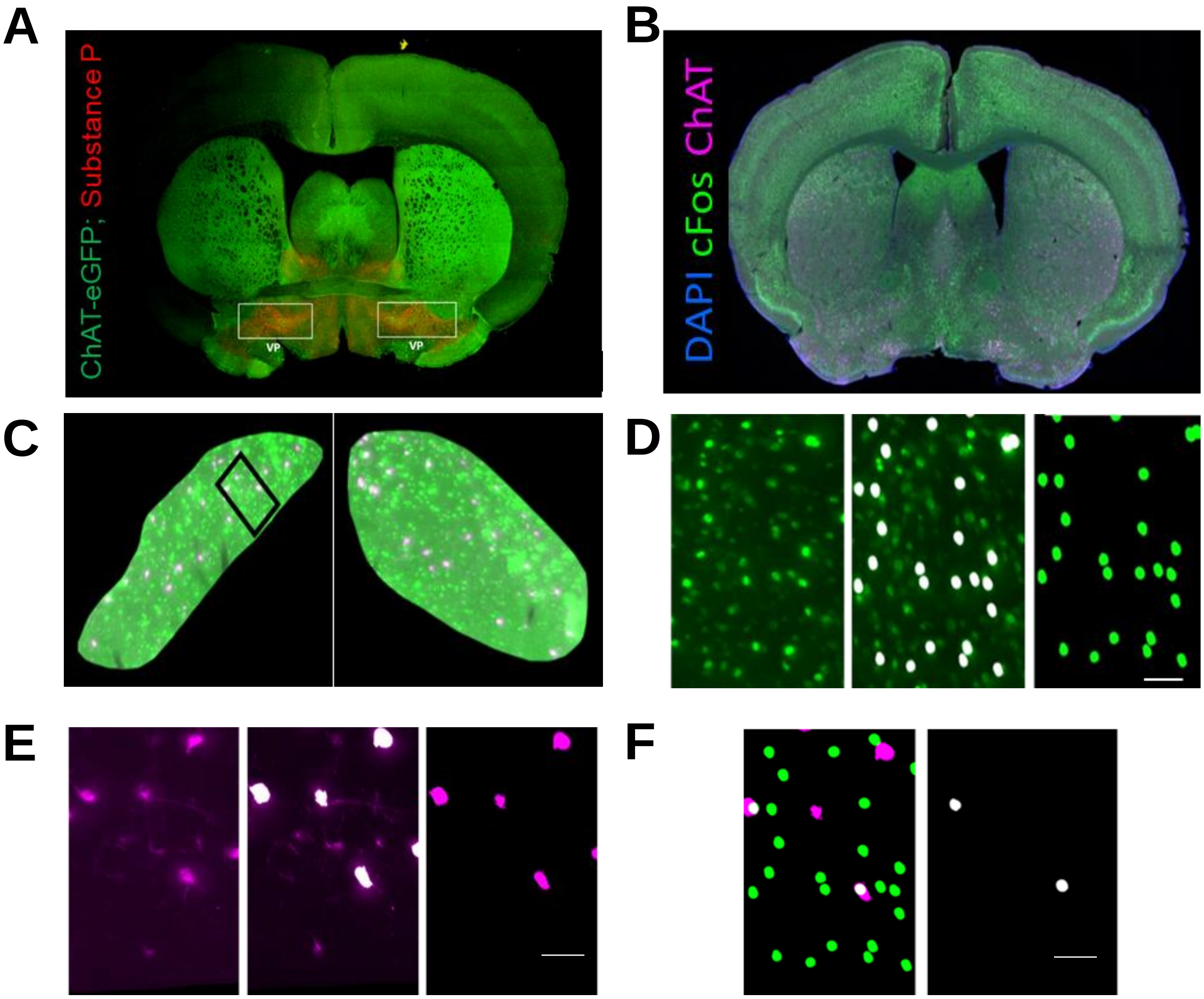

bioRxiv preprint doi: https://doi.org/10.1101/2023.10.06.561261; this version posted October 10, 2023. The copyright holder for this preprint (which was not certified by peer review) is the author/funder. This article is a US Government work. It is not subject to copyright under 17 USC 105 and is also made available for use under a CC0 license.

Fig S3

DIO-hM4Di APP odor Preference Test

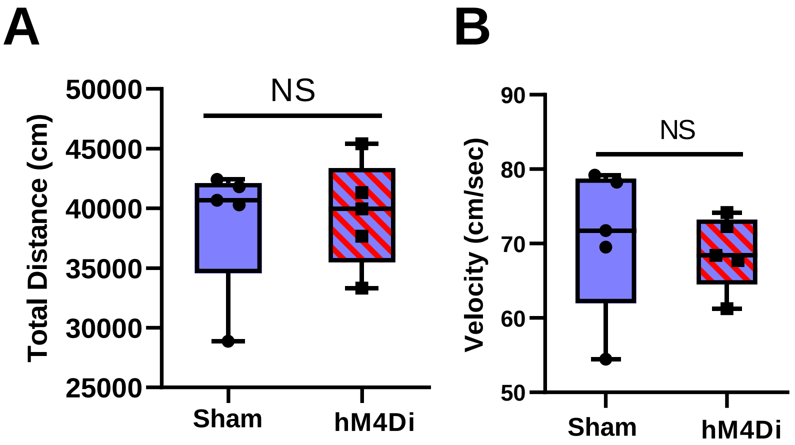

DIO-hM4Di AV odor Preference Test

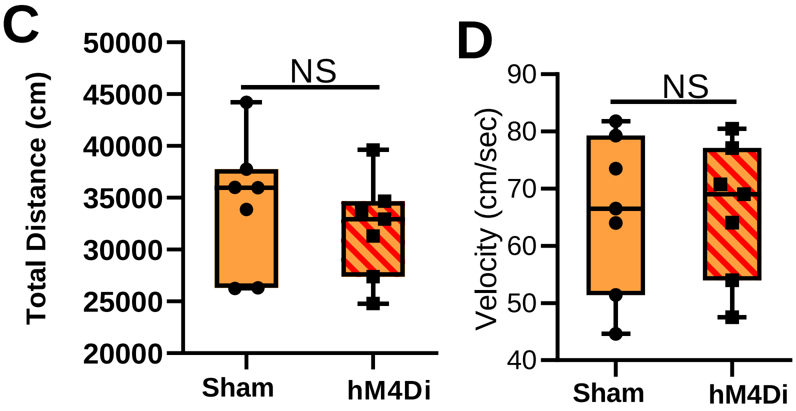

Fig S4

bioRxiv preprint doi: https://doi.org/10.1101/2023.10.06.561261; this version posted October 10, 2023. The copyright holder for this preprint (which was not certified by peer review) is the author/funder. This article is a US Government work. It is not subject to copyright under 17 USC 105 and is also made available for use under a CC0 license.

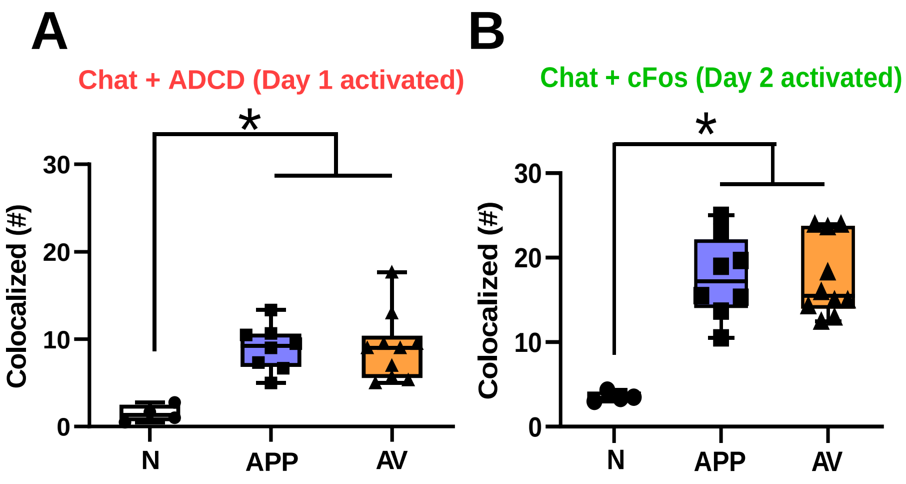

bioRxiv preprint doi: https://doi.org/10.1101/2023.10.06.561261; this version posted October 10, 2023. The copyright holder for this preprint (which was not certified by peer review) is the author/funder. This article is a US Government work. It is not subject to copyright under 17 USC 105 and is also made available for use under a CC0 license.

Fig S5

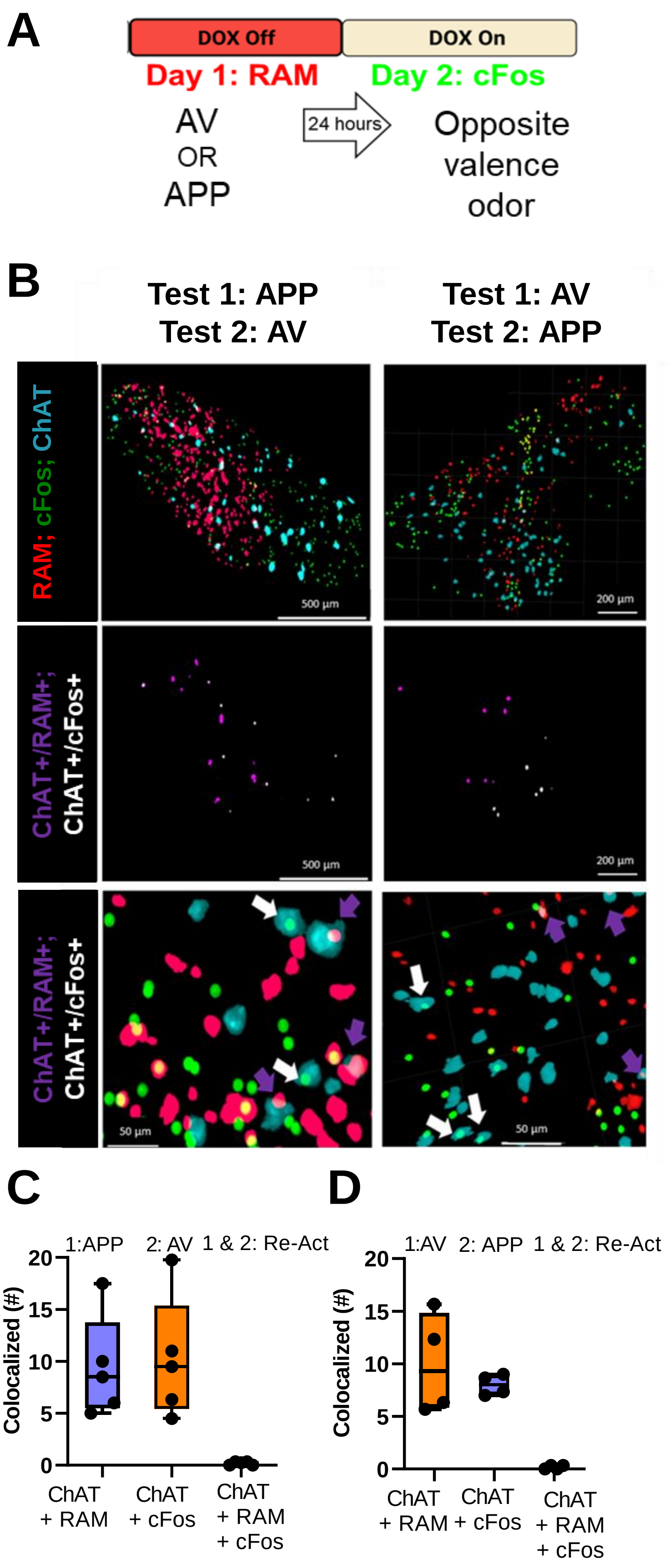

bioRxiv preprint doi: https://doi.org/10.1101/2023.10.06.561261; this version posted October 10, 2023. The copyright holder for this preprint (which was not certified by peer review) is the author/funder. This article is a US Government work. It is not subject to copyright under 17 USC 105 and is also made available for use under a CC0 license.

Fig S6

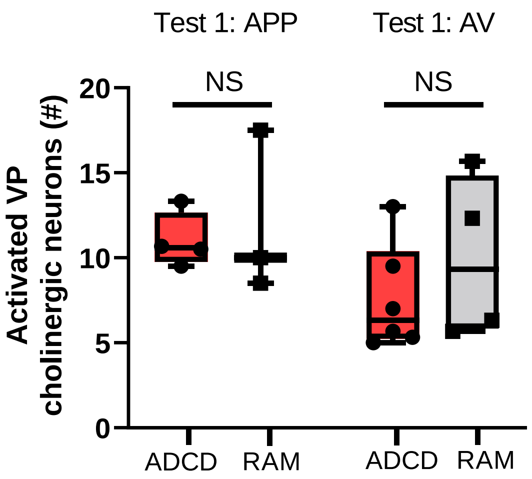

Fig S7
bioRxiv preprint doi: https://doi.org/10.1101/2023.10.06.561261; this version posted October 10, 2023. The copyright holder for this preprint (which was not certified by peer review) is the author/funder. This article is a US Government work. It is not subject to copyright under 17 USC 105 and is also made available for use under a CC0 license.

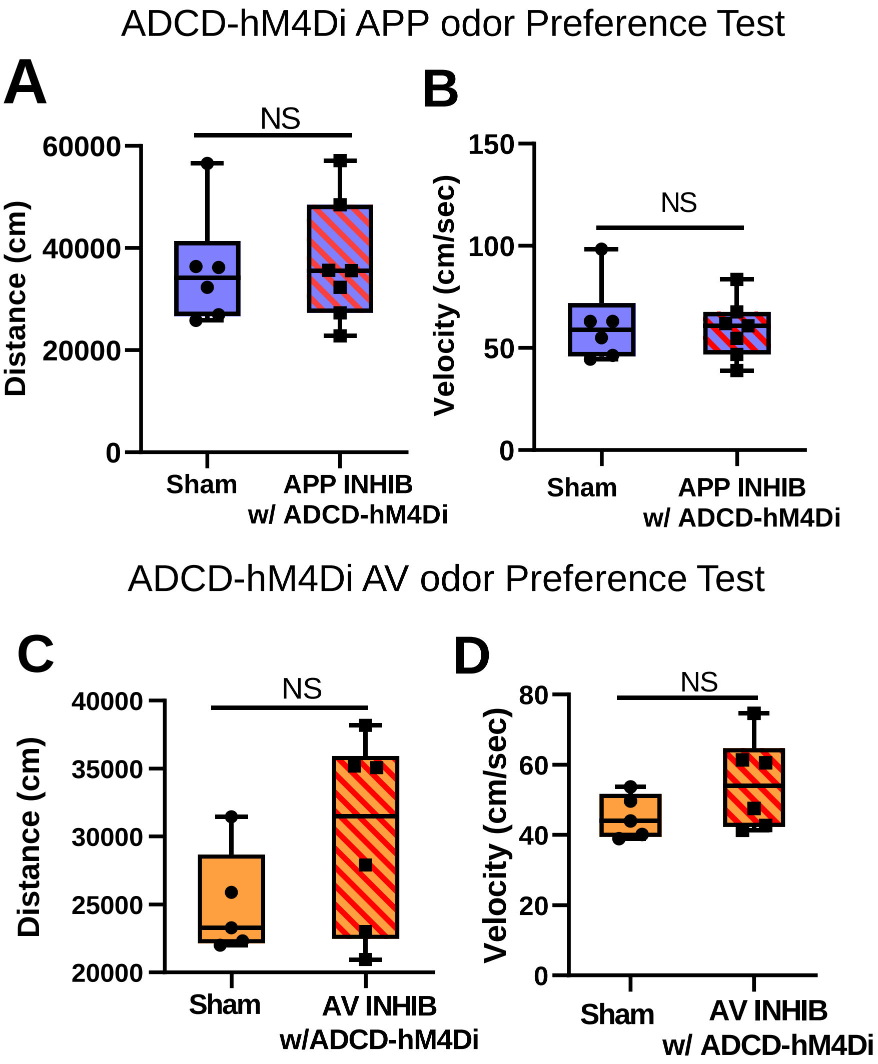

Fig S8

bioRxiv preprint doi: https://doi.org/10.1101/2023.10.06.561261; this version posted October 10, 2023. The copyright holder for this preprint (which was not certified by peer review) is the author/funder. This article is a US Government work. It is not subject to copyright under 17 USC 105 and is also made available for use under a CC0 license.

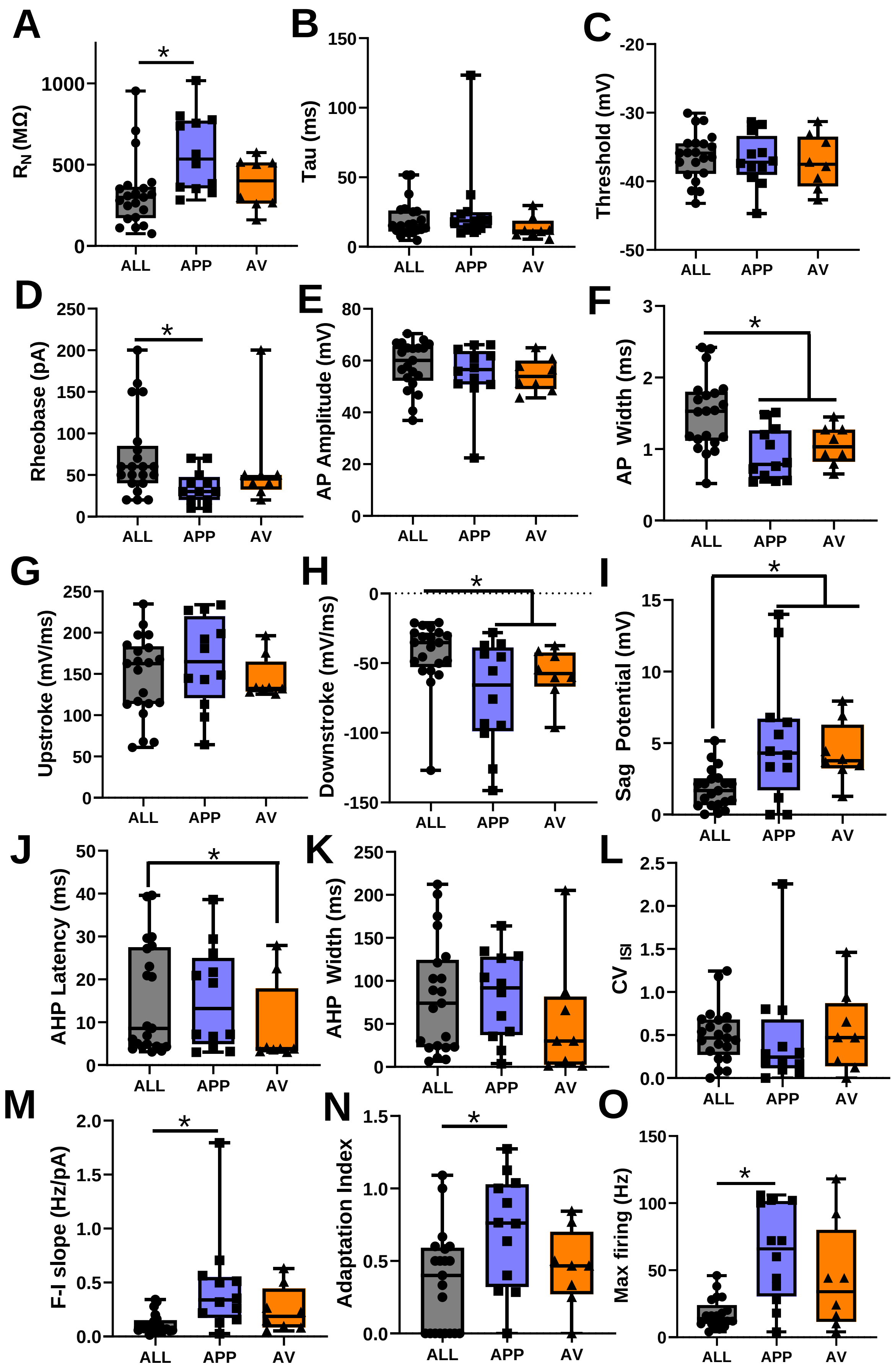

Fig S9

bioRxiv preprint doi: https://doi.org/10.1101/2023.10.06.561261; this version posted October 10, 2023. The copyright holder for this preprint (which was not certified by peer review) is the author/funder. This article is a US Government work. It is not subject to copyright under 17 USC 105 and is also made available for use under a CC0 license.

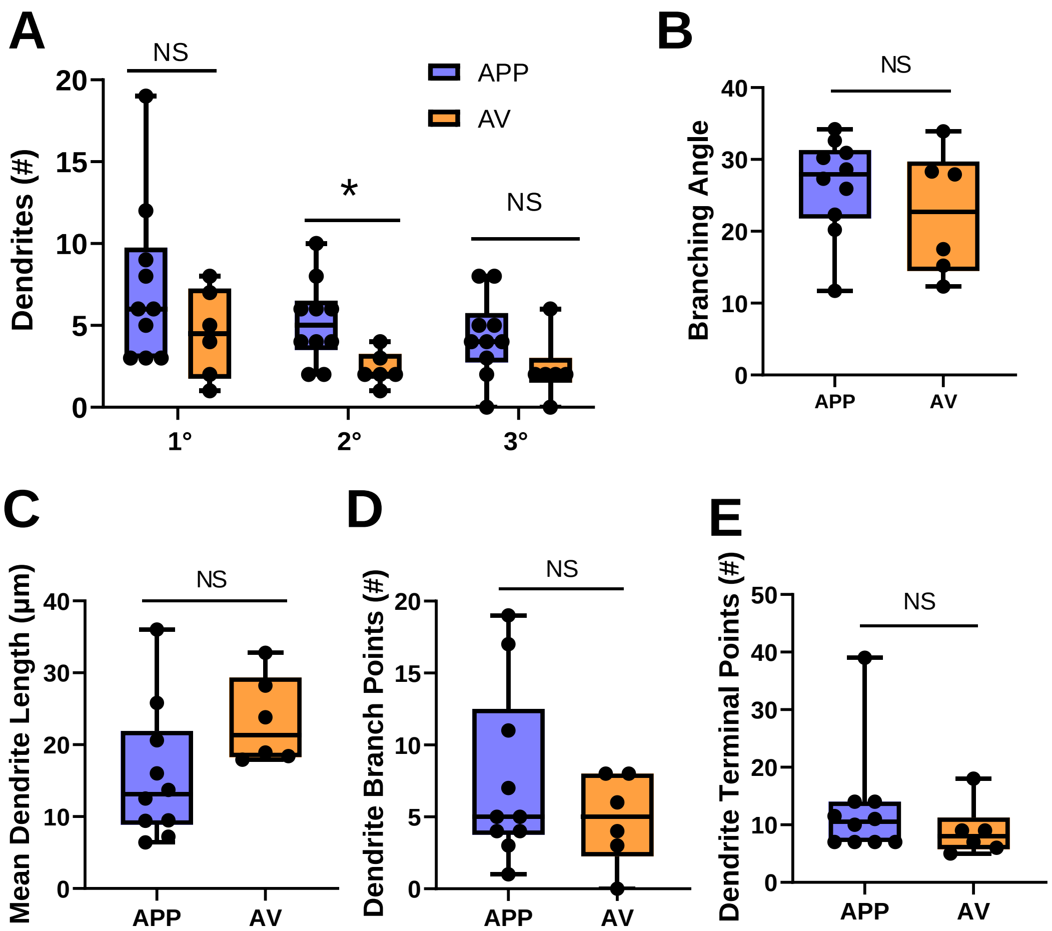

Supplement: 2 [file NIHPP2023.10.06.561261V1-supplement-1.pdf]
